# Association of vitamin E on the risk of ovarian cancer: a meta-analysis

**DOI:** 10.1042/BSR20193311

**Published:** 2019-12-23

**Authors:** Youxu Leng, Hairong Zhou, Fanjing Meng, Tian Tian, Jianying Xu, Fengjuan Yan

**Affiliations:** 1 Department of Gynecology, Longhua District Central Hospital, Shenzhen, 518110, Guangdong Province, China; 2 Department of General Medical Department, Longhua District Central Hospital, Shenzhen, 518110, Guangdong Province, China; 3 Department of Gynecology, Longhua District People’s Hospital, Shenzhen 518109, Guangdong Province, China; 4 Department of Gynecology, Dongguan City Tangxia Hospital, Dongguan 523710, Guangdong Province, China

**Keywords:** food, meta-analysis, ovarian cancer, supplement, vitamin E

## Abstract

Many researches were conducted to assess the association of vitamin E intake on the risk of ovarian cancer, with conflict results. The current meta-analysis of published observational studies aimed to investigate the effect of vitamin E intake on ovarian cancer risk. The summary relative risks (RRs) with corresponding 95% confidence intervals (CIs) were calculated to measure the effectiveness of vitamin E intake on ovarian cancer risk using a random-effects model. As a result, 14 studies including 4597 patients were identified. Eleven studies reported about total vitamin E intake, eight studies about vitamin E intake from food only and five studies about vitamin E intake from supplement only on the risk of ovarian cancer. Overall, the summary RRs on ovarian cancer risk was 0.95 (95%CIs = 0.78–1.16) in total vitamin E intake, 0.99 (95%CIs = 0.77–1.27) in vitamin E intake from food only and 0.82 (95%CIs = 0.54–1.25) in vitamin E intake from supplement only. Results in subgroup analyses by study design and geographic location were consistent with overall result. In conclusions, the findings of this meta-analysis suggested that high intake of vitamin E from food or vitamin E supplement had no significant effect on the risk of ovarian cancer.

## Introduction

Ovarian cancer is one of the most common malignant tumors in women, accounting for 2.5% of female cancers [1]. About 13,850 women die each year from ovarian cancer; this may be the main causes of cancer death in United States among females [2,3]. Furthermore, the 5-year survival rate with all types of ovarian cancer is about 47% [2,3]. Health education and diet prevention are important ways of preventing ovarian cancer. Vitamin E, also named as tocopherol, contains putative anti-cancer and anti-mutant substances that have long been considered to prevent cancer. It is also a well-known powerful antioxidant, which could protect cells against from oxidative DNA damage and mutagenesis, thereby preventing the onset of certain tumors [4,5]. Previous meta-analyses had been published to explore vitamin E intake on many tumors, such as lung cancer [6], esophageal cancer [7], uterine cervical neoplasm [8] and so on. To our attention, a lot of studies have investigated vitamin E intake from food or from supplement on the risk of ovarian cancer, resulting in inconsistent findings. The reasons for these differences included small sample size, low statistical capacity and/or clinical heterogeneity. To overcome the limitations of individual research and address these inconsistencies, we conducted this meta-analysis to provide a comprehensive overview.

## Materials and methods

### Search strategy

To collect studies that met the criteria for this meta-analysis, we reviewed all literature on the relationship between vitamin E and ovarian cancer risk. Literature searches were performed using Web of Science, EMBASE and PubMed databases (up to June 1, 2019). The following key words and subject terms were used in the search: “vitamin E” OR “vitamin*” OR “tocopherol” combined with “ovarian cancer” OR “ovarian tumor”. We also manually screened the bibliography of original research and review articles. The initial qualification is determined independently by the two reviewers. Disagreements between the reviewers are resolved by a third-party reviewer at a consensus meeting.

### Inclusion criteria and exclusion criteria

To be included in the meta-analysis, these studies must be: (1) case–control, cross-sectional or cohort studies; (2) patients were diagnosed as ovarian cancer; (3) report relative risks (RRs) and 95% confidence intervals (CIs) about vitamin E and ovarian cancer. When two or more articles report the same data, the most recently updated data will be considered. The two reviewers screened the titles and abstracts of the records retrieved from the literature. The full text of the potential related articles is independently searched and evaluated by two reviewers, and the differences in research qualifications are resolved by a third author.

The exclusion criteria were as follows: (1) case reports, conference abstracts, letters, editorials, reviews; (2) overlapping or duplicate studies; (3) irrelevant studies; (4) no available data of RRs and 95%CIs and (5) animal studies.

### Data extraction

Two investigators independently extracted these information from all eligible studies selected according to predefined criteria. Data were extracted about the last name of first author, age, study design, country, cases and participants, RR and 95%CI, sources of vitamin E (total vitamin E, vitamin E from food or vitamin E from supplement) and category of vitamin E on ovarian cancer risk.

### Statistical analysis

All statistical analyses were conducted using the STATA software V.12.0 (STATA Corp, College Station, Texas, U.S.A.). We conducted a meta-analysis of the relationship about vitamin E on ovarian cancer risk. Pooled results were expressed as RRs and 95%CIs with a random-effect model [9]. We used Cochran *Q* statistic and *I*² to assess variation and heterogeneity within and between studies [10]. The heterogeneity test was used to evaluate the null hypothesis that all studies evaluated the same effect. When the *Q* test *P* < 0.05 or *I*² > 50%, it is suggested that the study may be heterogeneous [11]. Sensitivity analysis was used to explore whether one single study had the essential effect on the overall RRs. To assess publication bias, visual observations using the Egger test [12] and the funnel plot [13] were used. When *P* < 0.05, the difference was statistically significant.

## Results

### Studies included in the meta-analysis

**Figure 1:**
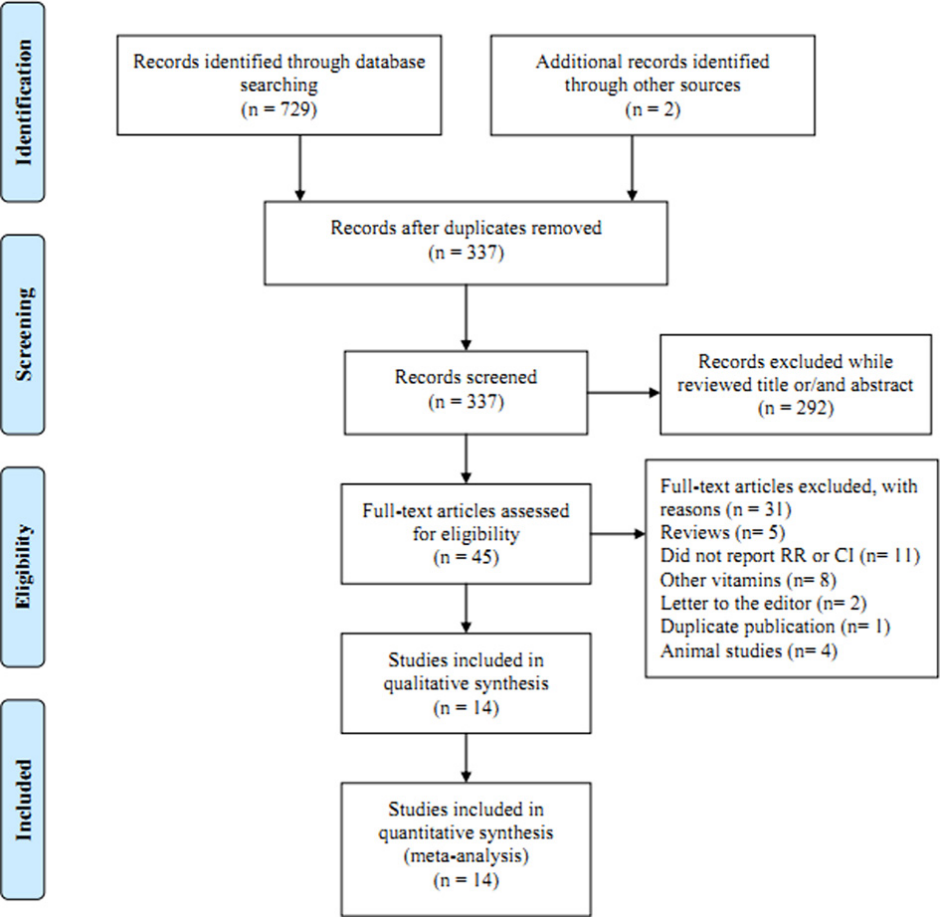
Flow chart of meta-analysis for exclusion/inclusion of studies

We identified 731 studies using electronic and manual retrieval methods, 394 of which were excluded because they were copies of other reports. Of the remaining 337 articles, 292 articles were excluded after review based on the title and abstract, and 45 were reviewed in full-text. Meanwhile, 31 articles were excluded due to reasons present in Figure 1. Finally, a total of 14 articles [14–27] involving 4597 cases met the inclusion criteria of this meta-analysis. Five articles were cohort design and the remaining nine articles were case–control design. All the studies come from North America except one from Asia. Table 1 summarizes some of the basic features of all included studies.

**Table 1:** Characteristics of each individual study included in our analysis

Study, Year	Design	Age	Participants, Cases	Country	Source of vitamin E	Category	RR (95%CI)
Chang et al., 2007	Cohort	<84	97,275, 280	United States	Total vitamin E intake	>207 versus ≤7 mg/d	1.46(0.76–2.79)
Cramer et al., 2001	PBCC	>50	1065, 549	United States	Total vitamin E intake	Q5 versus Q1	0.97(0.64–1.49)
Fairfield et al., 2001	Cohort	30–55	80,326, 301	United States	Total vitamin E intake From food	327 IU/day versus 5 IU/day 12 IU/day versus 5 IU/day	0.88(0.61–1.27) 1.52(1.04–2.21)
Fleischauer et al., 2001	HBCC	≥18	419, 168	United States	Total vitamin E intake From food From supplement	>43.5 versus <11.0 mg/d >10.5 versus <6.5 mg/d >30 mg/d versus none	0.59(0.30–1.15) 1.82(0.90–3.69) 0.43(0.26–0.72)
Gifkins et al., 2012	PBCC	>21	595, 205	United States	Total vitamin E intake From food From supplement	>114.9 versus <21.7 mg/d >11.6 versus <7.4 mg/d Yes versus no	1.03(0.59–1.78) 0.89(0.45–1.77) 1.63(1.02–2.63)
Kushi et al., 1999	Cohort	55–69	29,083, 139	United States	From food	>24.4 versus <6.2 mg/d	0.91(0.56–1.48)
McCann et al., 2001	HBCC	20–87	1921, 496	United States	From food	>9.4 versus ≤4.9 mg/d	0.58(0.38–0.88)
Pan et al. 2004	PBCC	20–76	2577, 442	Canada	From supplement	≥10 years versus never	0.49(0.30–0.81)
Salazar-Martinez et al., 2002	HBCC	20–79	713, 84	Mexico	Total vitamin E intake	≥9.4 versus ≤6.3 mg/d	1.60(0.88–2.95)
Silvera et al., 2006	Cohort	40–59	89,835, 264	Canada	Total vitamin E intake From food	>28 versus <17 mg/d >25 versus <17 mg/d	1.24(0.85–1.82) 0.87(0.57–1.31)
Terry et al. 2017	PBCC	20–79	1038, 406	United States	Total vitamin E intake From food From supplement	>25.8 versus <6.7 mg/d >9.1 versus <4.1 mg/d ≥13.5 mg/d versus never	0.91(0.61–1.37) 0.90(0.49–1.67) 0.86(0.61–1.20)
Thomson et al., 2008	Cohort	50–79	133,614, 451	United States	Total vitamin E intake From food From supplement	>403.2 versus <7.4 mg/d >9.4 versus ≤4.9 mg/d >200 mg/d versus never	1.22(0.89–1.66) 1.05(0.71–1.57) 1.12(0.86–1.45)
Tung et al., 2005	PBCC	45–75	1165, 558	United States	Total vitamin E intake	Q4 versus Q1	0.80(0.56–1.16)
Zhang et al., 2004	HBCC	18–75	906, 254	China	Total vitamin E intake	≥38.55 versus ≤23.40 mg/d	0.41(0.24–0.70)

Abbreviations: CI, confidence intervals; HBCC, hospital-based case–control study; PBCC, population-based case–control study; RR, relative risk.

### Total vitamin E intake and ovarian cancer risk

**Figure 2:**
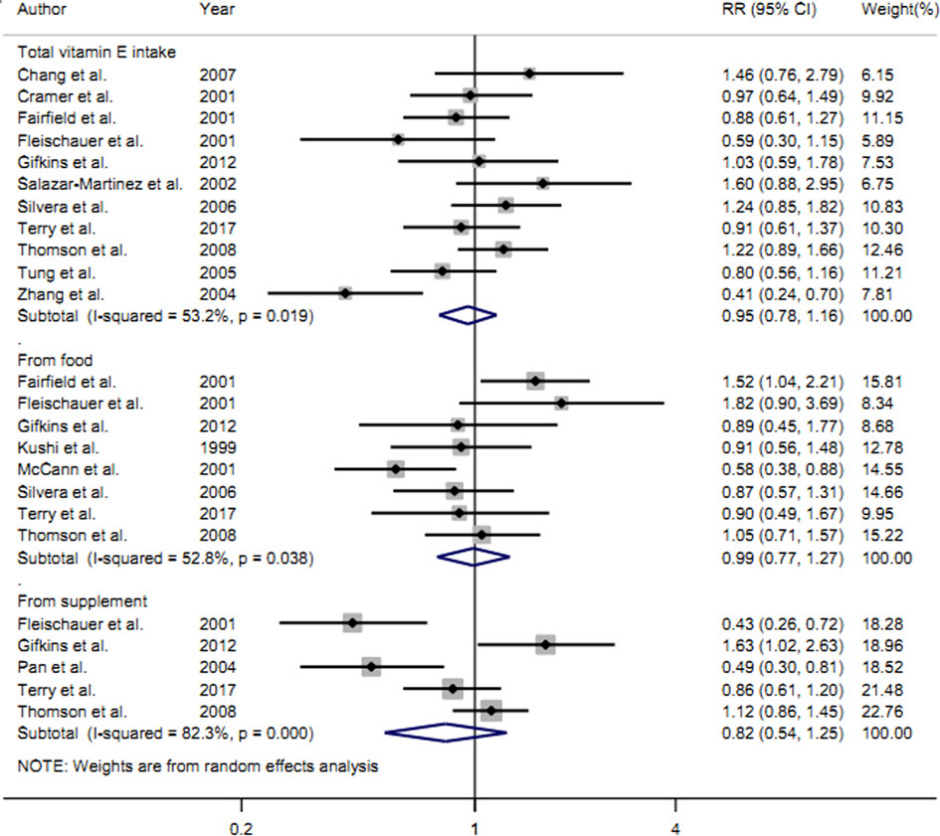
The forest plot about total vitamin E intake, vitamin E intake from food and vitamin E intake from supplement on ovarian cancer risk

Eleven studies [14–18,22–27] with 3520 cases reported total vitamin E intake on the risk ovarian cancer. Meta-analysis revealed that high category of total vitamin E intake had no significant effect on the risk of ovarian cancer (RRs = 0.95, 95%CIs = 0.78–1.16, *I*^2^ = 53.2%, *P*_for heterogeneity_ = 0.019) (Figure 2). Four studies were cohort design and seven studies were with case–control design. Subgroup analysis by study design showed that the summary RRs on ovarian cancer risk was 1.14 (95%CIs = 0.94–1.38, *I*^2^ = 0.0%, *P*_for heterogeneity_ = 0.417) in cohort studies and 0.84 (95%CIs = 0.64–1.11, *I*^2^ = 55.6%, *P*_for heterogeneity_ = 0.036) in case–control studies. Meanwhile, we further explore the association between ovarian cancer and geographic location. The results suggested that total vitamin E intake is not associated with the risk of ovarian cancer in North America (RRs = 1.02, 95%CIs = 0.88–1.19, *I*^2^ = 16.8%, *P*_for heterogeneity_ = 0.288).

### Vitamin E intake from food only and ovarian cancer risk

Eight studies [16–20,23–25] with 2430 cases about vitamin E intake from food only on ovarian cancer risk were included. Overall, the summary RRs on ovarian cancer risk was 0.99 (95%CIs = 0.77–1.27, *I*^2^ = 52.8%, *P*_for heterogeneity_ = 0.038) in vitamin E intake from food only (Figure 2). Four studies were cohort design and the remaining four studies were with case–control design. Subgroup analysis by study design showed high category of vitamin E intake from food only had no significant association on ovarian cancer risk either in cohort studies (RRs = 1.08, 95%CIs = 0.83–1.40, *I*^2^ = 35.9%, *P*_for heterogeneity_ = 0.197) or in case–control studies (RRs = 0.91, 95%CIs = 0.57–1.46, *I*^2^ = 60.8%, *P*_for heterogeneity_ = 0.054).

### Vitamin E intake from supplement only and ovarian cancer risk

Five studies [17,18,21,24,25] with 1672 cases reported vitamin E intake from supplement only on the risk of ovarian cancer. Overall, the summary RRs of vitamin E intake from supplement only on ovarian cancer risk was 0.82 (95%CIs = 0.54–1.25, *I*^2^ = 82.3%, *P*_for heterogeneity_ < 0.001).

### Publication bias and sensitivity analysis

**Figure 3:**
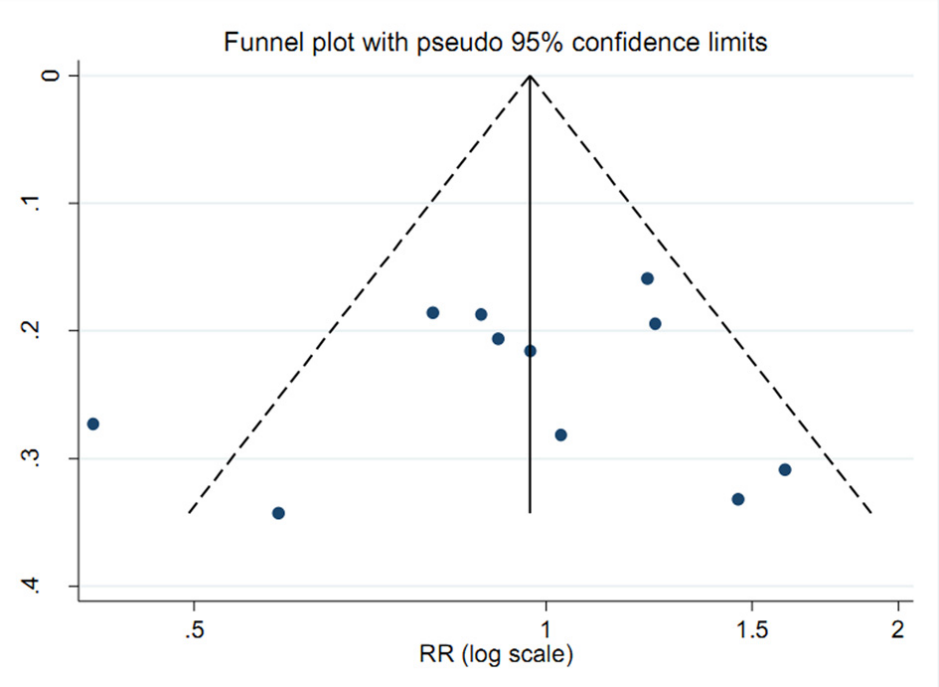
Funnel plot for the analysis of publication bias between total vitamin E intake and ovarian cancer risk

To assess publication bias, Begg’s test was conducted. After the analysis is complete, we did not detect any significant publication bias in total vitamin E intake (*P* = 0.620), in vitamin E intake from food only (*P* = 0.876) and in vitamin E intake from supplement only (*P* = 0.365). Figure 3 shows the funnel plot about total vitamin E intake and ovarian cancer risk. Sensitivity analyses indicated that no singly study had essential effect on the overall results.

## Discussion

Numerous of studies about vitamin E intake and ovarian cancer risk have been published, with conflicting results. We therefore conducted the present study to clarify whether high category of total vitamin E intake, vitamin E intake from food and vitamin E intake from supplement had some effective on the development of ovarian cancer. A total of 14 papers involving 4597 ovarian cancer cases were used in the present study. Pooled results suggested that high category of total vitamin E intake, vitamin E intake from food only and vitamin E intake from supplement only are not associated with the risk of ovarian cancer. Subgroup analyses by geographic location and study design in total vitamin E intake and subgroup analysis by study design in vitamin E intake from food only obtained consistent results with overall result. These results of the present study suggested to clinicians and researchers that there is no need to supplement vitamin E or intentionally eat more vitamin E.

Between-study heterogeneity existed in our study, which may affect the conclusions of the meta-analysis, although random effects model have been carried out. We used meta-regression to explore the potential heterogeneity. To our attention, geographic location may be a covariate for this high heterogeneity in total vitamin E intake. When we performed the subgroup analysis by geographic location, the *I*^2^ was reduced to 16.8% in North America populations and no analysis for Asia due to only one study from Asia. The result was not changed in North America. We did not detect any other influence factors on this high heterogeneity in total vitamin E intake, vitamin E intake from food and vitamin E intake from supplement.

Previous review by Koushik et al. [28] had explored the association about vitamin E intake on ovarian cancer risk from Pooling Project investigations of ovarian cancer risk. They concluded that consumption of vitamin E did not play a major role in ovarian cancer risk. Another review published by Crane et al. [29] concluded that no association was demonstrated for vitamin E intake on ovarian cancer risk while only included three papers. Results in the current meta-analysis were consistent with the previous reviews. Although our study did not obtain a positive result, we included more studies than the above-mentioned reviews. Furthermore, we also explored the association about total vitamin E intake, vitamin E intake from food and vitamin E intake from supplement on ovarian cancer risk, respectively. Even though, trial sequential analysis should be performed to see if more investigations were needed [30].

Some limitations should be stated in this meta-analysis. First, any literature-based review and meta-analysis are facing a major threat, namely reporting bias (only recruiting published English literature). Second, the sample size of some studies is relatively small. Third, almost all the included studies come from North America, and the results in our study are suitable for North America only. Therefore, to explore the relationship about vitamin E on the risk of ovarian cancer, more studies with other countries are warranted to further confirm this result. There is no doubt that the limitations mentioned above may affect our final conclusions. However, this meta-analysis also has its advantages. To the best of our knowledge, our meta-analysis is the comprehensive evidence to provide vitamin E intake on ovarian cancer risk. Similarly, compared with individual studies, our data on the relationship between vitamin E intake and ovarian cancer risk, due to the results of multiple independent analysis, improved statistical power and resolution, resulting in higher accuracy. However, given the limitations described above, further research is needed to address bias, confusion and opportunity.

## Conclusions

In conclusions, the findings of this meta-analysis indicated that high intake of vitamin E from food or vitamin E supplement had no significant effect on the risk of ovarian cancer. More studies are required to further explore these associations due to some limitations existed in our study.

## Abbreviations

CI: confidence interval
HBCC: hospital-based case–control study
PBCC: population-based case–control study
RR: relative risk
